# Targeting ketone body metabolism to treat fatty liver disease

**DOI:** 10.3389/jpps.2024.13375

**Published:** 2024-09-30

**Authors:** Sora Kwon, Reshani Jeyaratnam, Kyoung-Han Kim

**Affiliations:** ^1^ University of Ottawa Heart Institute, Ottawa, ON, Canada; ^2^ Department of Cellular and Molecular Medicine, Faculty of Medicine, University of Ottawa, Ottawa, ON, Canada; ^3^ Translational and Molecular Medicine, Faculty of Medicine, University of Ottawa, Ottawa, ON, Canada

**Keywords:** MASLD, ketone bodies, ketogenesis, dietary interventions, pharmacological interventions

## Abstract

Metabolic dysfunction-associated steatotic liver disease (MASLD) is a metabolic disorder marked by excessive accumulation of lipids within the liver. If untreated, this condition can progress to metabolic dysfunction-associated steatohepatitis (MASH), fibrosis, cirrhosis, and ultimately, hepatocellular carcinoma (HCC). Given the liver’s pivotal role in glucose and fatty acid metabolism, disruptions in these processes are commonly observed in MASLD. Ketone bodies, crucial energy metabolites primarily produced in the liver, are also closely related to the progression of MASLD. Recent studies have demonstrated that disrupted ketogenesis not only accompanies MASLD, but may also play a causal role in its development and progression. Moreover, activation of the ketogenic pathway has been suggested as a promising strategy for reducing excessive hepatic fat accumulation. This review focuses on the regulation of ketogenesis in MASLD, emphasizing the significance of dietary and pharmacological interventions as potential therapeutic approaches to treat fatty liver disease.

## Introduction

Metabolic dysfunction-associated steatotic liver disease (MASLD), formerly known as non-alcoholic fatty liver disease (NAFLD), is a prevalent chronic liver disease [1, 2], globally affecting human health with an estimated prevalence of 32% [3]. This condition is characterized by increased fat accumulation within the liver, compromising its function. The prolonged accumulation of hepatic fat in MASLD can lead to severe conditions, such as metabolic dysfunction-associated steatohepatitis (MASH), cirrhosis, and hepatocellular carcinoma (HCC). This progression is driven by lipotoxicity, leading to increased hepatic oxidative stress and the development of MASH [4]. Concurrently, increases in free fatty acid uptake and oxidative stress activate resident liver macrophages, which promote inflammation through various signaling pathways, including Toll-like receptor (TLR) 4-mediated production of pro-inflammatory cytokines [5, 6]. As the liver attempts to repair itself amid heightened inflammation, fibrosis emerges, characterized by the accumulation of extracellular matrix proteins, tissue scarring and immune cell infiltration [7, 8]. This persistent tissue scarring and immune activity eventually culminate into cirrhosis, marked by hepatocyte apoptosis [9] and impaired regenerative capacity [10]. Additionally, the elevated pro-inflammatory cytokine TNF has been associated with tumor promotion, as it stimulates hepatocyte proliferation, which can trigger the development of HCC [11]. As MASLD and its pathological progression arise from complex interactions of various factors affecting a broad spectrum of individuals, numerous studies have focused on elucidating the mechanisms driving the progression of this disease and developing effective therapeutic strategies.

Nevertheless, current treatment approaches for fatty liver disease, aside from lifestyle modifications such as weight management, dietary interventions, and exercise, are relatively limited. Insulin sensitizers, lipid-lowering medications, and antioxidants have been tested, but have not proven effective. Notably, drugs used for type 2 diabetes, such as metformin and sodium-glucose cotransporter-2 inhibitors (SGLT2i), have shown efficacy in treating fatty liver disease [12–14]. It remains unclear, though, whether the beneficial effects of these drugs on fatty liver disease are due to direct targeting of the liver function or are indirectly achieved through improved glucose homeostasis. Recently, the U.S. Food and Drug Administration approved resmetirom (Rezdiffra), a thyroid hormone receptor β (THRβ) agonist, as the first drug to directly target the liver for the treatment of MASH and moderate-to-advanced hepatic fibrosis. However, only 20%–30% of patients have shown improvement in key liver pathology indicators, and the long-term safety of resmetirom has not yet been assessed in clinical trials [15]. Therefore, the need to identify novel therapeutic targets for treating fatty liver disease remains a pressing and unmet challenge.

### Dysregulated ketone body metabolism in fatty liver disease

Metabolic remodelling is a molecular and cellular hallmark in fatty liver diseases, which includes alterations in *de novo* lipogenesis, hepatic very-low-density lipoprotein secretion and lipoprotein metabolism, and gluconeogenesis [16]. Another notable change is the dysregulation of ketone body metabolism. In the early stage of fatty liver disease like simple steatosis, an increase in plasma ketone bodies is often observed as a result of the liver converting excessive fatty acids into ketone bodies to alleviate metabolic stress [17, 18]. However, as MASLD advances to more severe stages like MASH, levels of plasma ketone bodies in patients decrease [19]. This decline is attributed to impaired ketogenesis, a process of synthesizing water-soluble ketone bodies, such as β-hydroxybutyrate (BHB), acetoacetate (AcAc), and acetone, primarily in the liver, as fasting-induced ketosis is significantly reduced in humans with MASLD [20, 21]. In addition, the rate of ketogenesis, specifically the production of BHB and not AcAc, is negatively associated with the degree of hepatic triglyceride content [20]. Impaired ketogenesis in severe MASLD has also been consistently observed in both preclinical mouse models and humans [22, 23].

Ketone bodies are primarily generated in the liver during glucose-deprived conditions. Acetyl-CoA, mainly derived from fatty acids through beta-oxidation, undergoes a series of enzymatic reactions within the mitochondria. These reactions involve acetoacetyl-CoA thiolase (ACAT1), 3-hydroxy-3 methyglutaryl-CoA synthase 2 (HMGCS2) and HMG-CoA lyase (HMGCL), generating AcAc as a primary ketone body metabolite. AcAc is then further converted to BHB by β-hydroxybutyrate dehydrogenase (BDH1) [24–26]. Among these critical enzymes in the ketogenic pathways, HMGCS2 is notably implicated in dysregulated ketogenesis in fatty liver disease. In mice with high-fat diet (HFD)-induced MASLD, the fasting-induced increases in HMGCS2 transcript and protein are largely abolished [22]. Similarly, HMGCS2 expression is suppressed with more advanced steatotic stages, such as cirrhosis and HCC [27, 28].

Importantly, dysregulated ketogenesis is not simply an outcome but plays a causal role in the development of fatty liver disease. In infants, deficiencies in HMGCS2 or HMGCL lead to hepatomegaly and hepatic steatosis [29–31]. Consistently, postnatal mice lacking *Hmgcs2* gene spontaneously develop fatty liver disease [22, 32]. The impaired hepatic ketogenic conduit by Hmgcs2 ablation causes excessive accumulation of acetyl-CoA [32, 33]. This, in turn, enhances *de novo* lipogenesis, hepatic glucose production, and acetylation of mitochondrial proteins, which collectively contribute to steatosis and metabolic dysfunctions in the liver. In addition, altered hepatic ketogenesis and ketone body metabolism contribute to the progression of fatty liver disease by modulating inflammation and fibrosis. For instance, ketogenic insufficiency induced by antisense oligonucleotide (ASO)-mediated *Hmgcs2* knockdown in HFD-fed adult mice results in not only elevated hepatic triacylglycerol concentrations but also inflammation and injury with macrophage accumulation in the liver, characteristics of MASH [34–36]. Also, disturbance in hepatocyte-macrophage ketone body communication, specifically via AcAc (not BHB), leads to hepatic fibrosis by activating hepatic stellate cells [37]. Furthermore, hepatic deletion of monocarboxylate transporter 1 (MCT1, encoded by *Slc16a1*), one of the main transporters of ketone bodies [38], exacerbates hepatic steatosis in female mice [39], although it is unclear whether this aggravation of the fatty liver is mediated by impaired ketone body transport. Disruptions in key regulators of ketogenesis, including hormones such as insulin and glucagon and transcriptional regulators like PPARα and mTORC1 [40], also contribute to the development of fatty liver disease. For example, *PPARα* knockout mice, which exhibit impaired ketogenesis with decreased ketogenic enzymes, Hmgcs2 and Bdh1, develop hepatic steatosis [41–43]. Additionally, mTORC1, which suppresses Hmgcs2 expression and ketogenesis by inhibiting the transcriptional activity of PPARα [44], is frequently activated in fatty liver disease [45]. Collectively, these findings underscore the critical role of ketone body metabolism in MASLD development and progression. Investigations into key enzymes and regulators, such as HMGCS2, BDH1, PPARα, and mTORC1, highlight the intricate interplay between ketone body metabolism and fatty liver disease.

### Targeting ketone body metabolism to treat fatty liver disease

Ketone bodies primarily serve as alternative energy fuels in extrahepatic tissues - such as the heart, skeletal muscle, and brain - during various developmental and physiological conditions, including neonatal development, pregnancy, starvation, and exercise. Importantly, the multifaceted roles of ketone bodies in metabolic health have been extensively studied. They mediate cellular signaling via G-protein receptors (i.e., GPR41, GPR43 and GPR109A) and epigenetic gene regulation through post-translational modifications (PTMs), including histone modifications, such as lysine acetoacetylation and β-hydroxybytyrylation [46–48]. These mechanisms collectively exert anti-inflammatory, antioxidative and antifibrotic effects [49–53].

**FIGURE 1:**
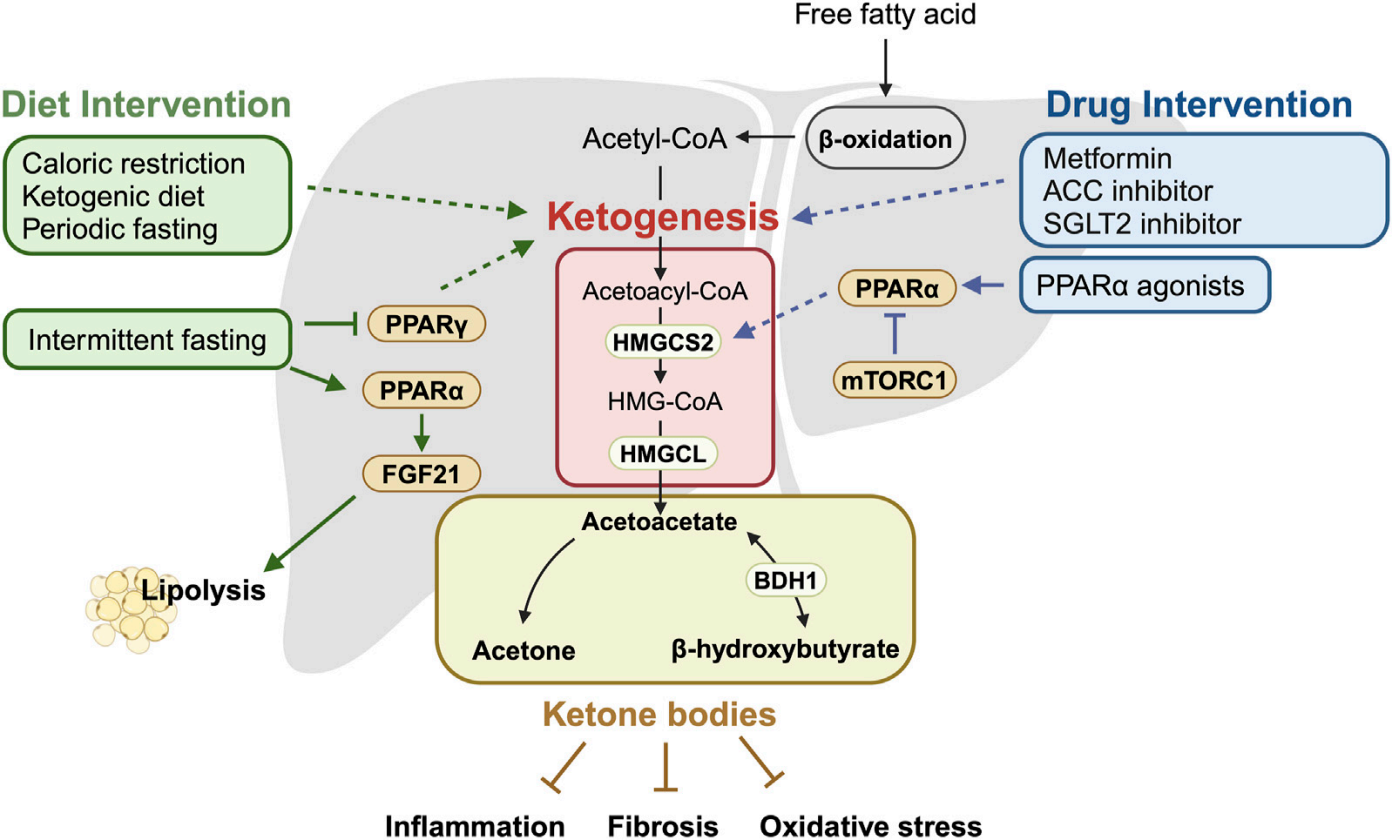
Summary of ketogenesis mechanisms in relation to dietary and pharmacological interventions. This schematic illustrates the key enzymes and pathways involved in ketogenesis and how they are modulated by dietary and pharmacological interventions. It highlights the impact of these interventions on the ketogenic pathway, leading to increased production of ketone bodies, which are crucial for managing MASLD. Activated ketogenesis helps reduce liver fat accumulation and inhibit fatty liver disease progression factors, such as inflammation, fibrosis, and oxidative stress. Solid arrows indicate the direction of regulatory effect, while dotted arrows represent effects that are known but not fully understood. The schematic was created with BioRender.com.

It is noteworthy that elevations in ketogenesis and the administration of ketone bodies can provide significant benefits against the development and progression of fatty liver disease, underscoring the substantial health implications of ketone bodies (Figure 1). Specifically, activating ketogenesis through *Hmgcs2* overexpression improves HFD-induced MASLD in mice and reduces lipid accumulation in HepG2 cells [22]. Concurrently, *Bdh1* overexpression in the liver ameliorates hepatic fibrosis, inflammation and apoptosis in *db/db* mice [54]. In addition, the exogenous administration of AcAc reduces hepatic fibrosis in mice fed a fibrogenic diet [37], while BHB supplementation lessens liver injury and exerts anti-inflammatory effects through the down-regulation of the NLRP3 inflammasome [55–57]. Similarly, dietary supplementation with ketone esters decreases MASLD and inflammation, along with a reduction in the expression of profibrotic and proinflammatory genes, such as *Col1a1* and *Pdgfb* [58, 59]. These findings emphasize the potential therapeutic avenues for addressing MASLD and its progression by targeting ketone body metabolism. There is growing interest in utilizing dietary and pharmacological interventions to enhance ketogenesis for treating hepatic steatosis and its progression, as detailed further below.

#### Dietary interventions

As ketogenesis has emerged as a potent target for MASLD treatment, dietary interventions that influence ketone body metabolism, such as nutritional interventions and fasting regimens, offer promising approaches for managing MASLD. Indeed, besides various positive effects on health and lifespan, nutritional interventions have demonstrated promising therapeutic impacts on MASLD with decreased hepatic triglyceride content in mice and reduced body fat and inflammation markers in humans [60, 61]. Notably, nutritional interventions, such as caloric restriction (10%–40% reduction) and ketogenic diets, effectively elevate blood ketone body levels and enhance their transport and utilization in both rodents and humans [62–65]. Specifically, caloric restriction, which entails a significant reduction in daily calorie intake, has been shown to decrease hepatic fat content [66], thereby reversing hepatic steatosis in obese rodents with metabolic diseases [60]. In MASLD patients, caloric restriction leads to reductions in fatty liver index and ALT values [67], indicating potential therapeutic benefits. Furthermore, the ketogenic diet, characterized by limited carbohydrate intake, stimulates the mobilization of fatty acids, leading to weight loss in humans and mice. It also effectively increases their blood ketone body levels while improving plasma glucose and triglycerides as well as insulin sensitivity in MASLD patients. A low-carbohydrate ketogenic diet significantly reduces intrahepatic triglyceride levels by 43.8% and alleviates hepatic inflammation and fibrosis in MASLD patients [65, 68, 69]. Consistently, ketogenic diets decrease the expression of genes involved in fatty acid synthesis while upregulating those involved in fatty acid oxidation [70–73]. These beneficial effects of ketogenic diets in the liver are mediated through hepatic fibroblast growth factor 21 (FGF21) as a regulator of the ketotic state [74, 75]. Together, these findings suggest that nutritional interventions are effective strategies for treating MASLD by promoting ketone body metabolism. However, some studies have noted that a ketogenic diet may induce hepatic steatosis, increase inflammation, and promote cellular senescence in mice [64, 76, 77]. Such discrepancies among different studies may potentially be attributed to variations in dietary composition, particularly the fat content, as well as differences in diet duration and the ages of subjects or participants. This underscores the need to carefully evaluate the potential adverse effects of ketogenic diets and understand their underlying mechanisms.

Fasting interventions, such as intermittent fasting and time-restricted feeding, which involve alternating periods of fasting and refeeding [78, 79], are effective in promoting cyclic ketogenesis, thereby potentially improving MASLD [80, 81]. Various intermittent fasting (IF) regimens, such as time-restricted feeding, alternate-day fasting, 2:1 IF, and 5:2 IF, have been shown to improve steatosis by downregulation of PPARγ, a transcription factor implicated in triglyceride homeostasis and activation of fatty acid oxidation via PPARα, in high-fat-fructose induced MASH rat models [82] and HFD-induced MASLD mice [81, 83–85]. Notably, IF also activates the hepatic autophagy-lysosome pathway, reducing hepatic lipid accumulation [84] while diminishing hepatic inflammation and fibrosis through decreased expression of IL-6 and TNFα, thereby mitigating MASH progression [81, 83, 84]. Furthermore, IF has proven effective in humans, reducing intrahepatic triglyceride content by 8.3% [86]. Collectively, these studies highlight that nutritional and fasting interventions can serve as effective therapeutic approaches for MASLD via activating ketogenesis.

#### Pharmacological interventions

Several pharmacological candidates have shown potential for improving fatty liver disease outcomes by affecting ketone body metabolism. These include Metformin, PPARα agonist (Fibrates), ACC (Acetyl-CoA carboxylase) inhibitors and sodium-glucose cotransporter 2 (SGLT2) inhibitors [14, 87, 88].

Metformin (1, 1-dimethylbiguanide hydrochloride) has demonstrated potential in inhibiting the progression of MASLD. Clinical studies have indicated that metformin treatment in patients with MASLD improves liver function with reductions in hepatic fat accumulation and inflammation [89, 90]. By decreasing hepatic gluconeogenesis, metformin leads to reduced blood glucose levels, which in turn suppresses the activation of lipogenic pathways and promotes hepatic ketogenesis in rat liver [91]. It has also been shown that metformin induces fasting-mimicking metabolic modification, including ketogenesis, in humans [92]. However, the specific molecular mechanism by which metformin affects hepatic ketogenesis remains unclear, and it is unknown whether the metabolic therapeutic effects of metformin are mediated through ketone bodies.

PPARα agonists, such as fibrates, play a crucial role in regulating hepatic lipid metabolism. They have been shown to upregulate the expression of genes involved in fatty liver oxidation and lipoprotein metabolism, potentially contributing to increased ketogenesis [93, 94]. By enhancing these processes, fibrates could improve liver function and reduce hepatic fat accumulation in patients with fatty liver disease. Although fenofibrate has demonstrated efficacy in improving indicators of metabolic syndrome, blood sugar levels, and hepatic function tests in clinical investigations, it has not yielded significant improvement in liver histology, including steatosis score, inflammation grade, and fibrosis stage. To address these limitations, selective PPARα modulators like Pemafibrate have been developed, which offer improved efficacy and safety profiles. Specifically, Pemafibrate has been shown to ameliorate markers of liver inflammation and fibrosis in patients with MASLD [95, 96].

Acetyl-CoA carboxylase (ACC) is a pivotal enzyme in fatty acid synthesis, catalyzing the conversion of acetyl-CoA to malonyl-CoA, a crucial step in hepatic *de novo* lipogenesis. Owing to its central role in lipid metabolism, ACC has emerged as a promising target for therapeutic intervention in fatty liver disease. Numerous studies have demonstrated that inhibition of ACC can effectively reduce fatty acid synthesis and, consequently, decrease hepatic lipid accumulation [97]. For example, Firsocostat (GS-0976), a liver-targeted small molecule allosteric inhibitor of ACC1/2, improves MASH in both preclinical and clinical studies [98]. Additionally, another ACC1/2 inhibitor PF-05221304, either alone or in combination with a DGAT2 (diacylglycerol O-acyltransferase 2) inhibitor, significantly reduces hepatic steatosis in patients with MASLD [99]. Furthermore, it has been shown that a small molecule IMA-1, which interrupts the arachidonate 12-lipoxygenase (ALOX12)-ACC1 interaction, decreases hepatic lipid accumulation and lowers inflammation and fibrosis in mice and macaques, addressing multiple key features of MASH [100]. Notably, a single oral dose of MK-4074, a liver-specific ACC1/2 inhibitor, increases plasma ketone bodies in mice and humans within 8 h [101], suggesting its strong ketogenic potential. Similarly, the observation that Firsocostat can increase BHB in non-hepatic cells further supports the conserved ketogenic action of ACC inhibition [102]. However, the implications of ketone bodies in ACC inhibitor-mediated hepatic protection have not been explored.

Another class of drugs that have shown promise in the context of ketogenesis and MASLD is the SGLT2 inhibitors, commonly used in the treatment of type 2 diabetes [103]. These drugs increase urinary excretion of glucose by the kidney, thereby reducing blood glucose levels. Beyond their primary use, SGLT2 inhibitors offer therapeutic benefits for MASLD by modulating key metabolic pathways. They promote lipolysis, stimulate mitochondrial biogenesis and autophagy, and reduce lipogenesis, oxidative stress, and fibrogenesis [104, 105]. Meta-analyses have also shown that SGLT2 inhibitors can reduce hepatic enzymes (e.g., ALT and AST), hepatic fat contents, and Fibrosis-4 (FIB-4) levels, suggesting they alleviate MASLD and its progression to MASH [106]. Notably, it is well known that treatment with SGLT2 inhibitors is associated with higher plasma ketone body levels in patients [104, 105]. While the exact mechanism linking SGLT2 inhibitors and ketogenesis is not fully understood [107], it has been suggested that a metabolic shift from glucose to fatty acids induced by SGLT2 inhibitors underlies ketogenesis [104]. Nevertheless, it remains unclear whether the salutary actions of SGLT2 inhibitors against MASLD are mediated by promoting ketogenesis or through SGLT2-independent actions, as observed in the failing heart [108]. Future studies are required to uncover the therapeutic mechanism of SGLT2 inhibitors for MASLD.

Additionally, Pimozide, which blocks skeletal muscle ketone oxidation, increases plasma ketone bodies and improves hyperglycemia [109], yet its effects on fatty liver disease remain unknown. Rapamycin, which inhibits mTORC1, the negative modulator of hepatic ketogenesis, also increases plasma ketone bodies [44]. However, due to its intricate actions in global metabolism and crosstalk with several pathways [110], targeting the mTOR pathway to treat fatty liver disease presents challenges. It is noteworthy that these pharmacological agents appear to promote ketogenesis indirectly, including through transcriptional activation and modulation of metabolic fluxes. The development of drug candidates that directly target ketogenic enzymes and their roles in treating fatty liver disease hold significant interest.

## Discussion

In this review, we aim to summarize the current understanding of the potential role of ketogenesis as a critical player in the treatment of fatty liver disease, utilizing both dietary and drug interventions (Figure 1). The contributions of ketogenesis and ketone bodies in MASLD treatment are promising, yet further investigation is warranted to determine the extent to which the beneficial effects result from ketogenesis itself [22], the use of ketone bodies as fuel, or the cellular actions of ketone bodies as signaling molecules, or a combination of these processes [49]. In addition, careful consideration of several factors is required when evaluating treatment options that promote ketogenesis. For instance, ketoacidosis, a life-threatening complication of diabetes, has been reported as a potential side effect of both SGLT2 inhibitors [111] and ketogenic diets [112], though the underlying mechanisms are not fully understood. Furthermore, variations in the effects of ketogenic diets and intermittent fasting due to differences in sex and age have been observed [113, 114], as these factors are also known to impact ketone body metabolism [115]. Consequently, further investigation is essential to safely and effectively leverage ketone body metabolism for the treatment of fatty liver disease.

## References

[1] Eslam M Sanyal AJ George J Sanyal A Neuschwander-Tetri B Tiribelli C MAFLD: a consensus-driven proposed nomenclature for metabolic associated fatty liver disease. Gastroenterology (2020) 158:1999–2014.e1. 10.1053/j.gastro.2019.11.312 32044314

[2] Rinella ME Sookoian S. From NAFLD to MASLD: updated naming and diagnosis criteria for fatty liver disease. J Lipid Res (2024) 65:100485. 10.1016/j.jlr.2023.100485 38103785 PMC10824973

[3] Riazi K Azhari H Charette JH Underwood FE King JA Afshar EE The prevalence and incidence of NAFLD worldwide: a systematic review and meta-analysis. The Lancet Gastroenterol and Hepatol (2022) 7:851–61. 10.1016/S2468-1253(22)00165-0 35798021

[4] Feldstein AE Werneburg NW Canbay A Guicciardi ME Bronk SF Rydzewski R Free fatty acids promote hepatic lipotoxicity by stimulating TNF-α expression via a lysosomal pathway. Hepatology (2004) 40:185–94. 10.1002/hep.20283 15239102

[5] Sharifnia T Antoun J Verriere TGC Suarez G Wattacheril J Wilson KT Hepatic TLR4 signaling in obese NAFLD. Am J Physiology-Gastrointestinal Liver Physiol (2015) 309:G270–278. 10.1152/ajpgi.00304.2014 PMC4537925 26113297

[6] Slevin E Baiocchi L Wu N Ekser B Sato K Lin E Kupffer cells: inflammation pathways and cell-cell interactions in alcohol-associated liver disease. The Am J Pathol (2020) 190:2185–93. 10.1016/j.ajpath.2020.08.014 32919978 PMC7587925

[7] Sanchez JI Parra ER Jiao J Solis Soto LM Ledesma DA Saldarriaga OA Cellular and molecular mechanisms of liver fibrosis in patients with NAFLD. Cancers (Basel) (2023) 15:2871. 10.3390/cancers15112871 37296834 PMC10252068

[8] Bataller R Brenner DA. Liver fibrosis. J Clin Invest (2005) 115:209–18. 10.1172/JCI24282 15690074 PMC546435

[9] Ribeiro PS Cortez-Pinto H Solá S Castro RE Ramalho RM Baptista A Hepatocyte apoptosis, expression of death receptors, and activation of NF-κB in the liver of nonalcoholic and alcoholic steatohepatitis patients. Am J Gastroenterol (2004) 99:1708–17. 10.1111/j.1572-0241.2004.40009.x 15330907

[10] Dewhurst MR Ow JR Zafer G van Hul NKM Wollmann H Bisteau X Loss of hepatocyte cell division leads to liver inflammation and fibrosis. PLoS Genet (2020) 16:e1009084. 10.1371/journal.pgen.1009084 33147210 PMC7641358

[11] Park EJ Lee JH Yu GY He G Ali SR Holzer RG Dietary and genetic obesity promote liver inflammation and tumorigenesis by enhancing IL-6 and TNF expression. Cell (2010) 140:197–208. 10.1016/j.cell.2009.12.052 20141834 PMC2836922

[12] Targher G Mantovani A Byrne CD. Mechanisms and possible hepatoprotective effects of glucagon-like peptide-1 receptor agonists and other incretin receptor agonists in non-alcoholic fatty liver disease. The Lancet Gastroenterol and Hepatol (2023) 8:179–91. 10.1016/S2468-1253(22)00338-7 36620987

[13] Xing B Zhao Y Dong B Zhou Y Lv W Zhao W. Effects of sodium-glucose cotransporter 2 inhibitors on non-alcoholic fatty liver disease in patients with type 2 diabetes: a meta-analysis of randomized controlled trials. J Diabetes Invest (2020) 11:1238–47. 10.1111/jdi.13237 PMC7477503 32083798

[14] Farah S Nguyen T Kelsberg G Safranek S. Metformin for nonalcoholic fatty liver disease and nonalcoholic steatohepatitis. Am Fam Physician (2019) 99:262–3. 30763050

[15] Harrison SA Bedossa P Guy CD Schattenberg JM Loomba R Taub R A phase 3, randomized, controlled trial of resmetirom in NASH with liver fibrosis. N Engl J Med (2024) 390:497–509. 10.1056/NEJMoa2309000 38324483

[16] Bence KK Birnbaum MJ. Metabolic drivers of non-alcoholic fatty liver disease. Mol Metab (2021) 50:101143. 10.1016/j.molmet.2020.101143 33346069 PMC8324696

[17] Post A Garcia E van den Berg EH Flores‐Guerrero JL Gruppen EG Groothof D Nonalcoholic fatty liver disease, circulating ketone bodies and all-cause mortality in a general population-based cohort. Eur J Clin Invest (2021) 51:e13627. 10.1111/eci.13627 34120339 PMC9285047

[18] Satapati S Kucejova B Duarte JA Fletcher JA Reynolds L Sunny NE Mitochondrial metabolism mediates oxidative stress and inflammation in fatty liver. J Clin Invest (2015) 125:4447–62. 10.1172/JCI82204 26571396 PMC4665800

[19] Mannisto VT Simonen M Hyysalo J Soininen P Kangas AJ Kaminska D Ketone body production is differentially altered in steatosis and non-alcoholic steatohepatitis in obese humans. Liver Int (2015) 35:1853–61. 10.1111/liv.12769 25533197

[20] Fletcher JA Deja S Satapati S Fu X Burgess SC Browning JD. Impaired ketogenesis and increased acetyl-CoA oxidation promote hyperglycemia in human fatty liver. JCI Insight (2019) 4. 10.1172/jci.insight.127737 PMC6629163 31012869

[21] Lee S Bae J Jo DR Lee M Lee Y Kang ES Impaired ketogenesis is associated with metabolic-associated fatty liver disease in subjects with type 2 diabetes. Front Endocrinol (Lausanne) (2023) 14:1124576. 10.3389/fendo.2023.1124576 36896171 PMC9989459

[22] Asif S Kim RY Fatica T Sim J Zhao X Oh Y Hmgcs2-mediated ketogenesis modulates high-fat diet-induced hepatosteatosis. Mol Metab (2022) 61:101494. 10.1016/j.molmet.2022.101494 35421611 PMC9039870

[23] Mey JT Erickson ML Axelrod CL King WT Flask CA McCullough AJ β-Hydroxybutyrate is reduced in humans with obesity-related NAFLD and displays a dose-dependent effect on skeletal muscle mitochondrial respiration *in vitro* . Am J Physiology-Endocrinology Metab (2020) 319:E187–E195. 10.1152/ajpendo.00058.2020 PMC7468782 32396388

[24] McGarry JD Foster DW. Regulation of hepatic fatty acid oxidation and ketone body production. Annu Rev Biochem (1980) 49:395–420. 10.1146/annurev.bi.49.070180.002143 6157353

[25] Hegardt FG. Mitochondrial 3-hydroxy-3-methylglutaryl-CoA synthase: a control enzyme in ketogenesis. Biochem J (1999) 338(Pt 3):569–82. 10.1042/0264-6021:3380569 10051425 PMC1220089

[26] Laffel L. Ketone bodies: a review of physiology, pathophysiology and application of monitoring to diabetes. Diabetes Metab Res Rev (1999) 15:412–26. 10.1002/(sici)1520-7560(199911/12)15:6<412::aid-dmrr72>3.0.co;2-8 10634967

[27] Wang YH Liu CL Chiu WC Twu YC Liao YJ. HMGCS2 mediates ketone production and regulates the proliferation and metastasis of hepatocellular carcinoma. Cancers (Basel) (2019) 11:1876. 10.3390/cancers11121876 31779269 PMC6966636

[28] Ding R Chen T Zhang Y Chen X Zhuang L Yang Z. HMGCS2 in metabolic pathways was associated with overall survival in hepatocellular carcinoma: a LASSO-derived study. Sci Prog (2021) 104:003685042110317. 10.1177/00368504211031749 PMC10358623 34260294

[29] Rojnueangnit K Maneechai P Thaweekul P Piriyanon P Khositseth S Ittiwut C Expanding phenotypic and mutational spectra of mitochondrial HMG-CoA synthase deficiency. Eur J Med Genet (2020) 63:104086. 10.1016/j.ejmg.2020.104086 33045405

[30] Ago Y Otsuka H Sasai H Abdelkreem E Nakama M Aoyama Y Japanese patients with mitochondrial 3-hydroxy-3-methylglutaryl-CoA synthase deficiency: *in vitro* functional analysis of five novel HMGCS2 mutations. Exp Ther Med (2020) 20:1. 10.3892/etm.2020.9166 32952630 PMC7480138

[31] Urganç N Arapoglu M Evruke M Aydin A. A rare cause of hepatomegaly: 3-hydroxy-3-methylglutaryl coenzyme-a lyase deficiency. J Pediatr Gastroenterol Nutr (2001) 33:339–41. 10.1097/00005176-200109000-00022 11593134

[32] Arima Y Nakagawa Y Takeo T Ishida T Yamada T Hino S Murine neonatal ketogenesis preserves mitochondrial energetics by preventing protein hyperacetylation. Nat Metab (2021) 3:196–210. 10.1038/s42255-021-00342-6 33619377

[33] d'Avignon DA Puchalska P Ercal B Chang Y Martin SE Graham MJ Hepatic ketogenic insufficiency reprograms hepatic glycogen metabolism and the lipidome. JCI Insight (2018) 3. 10.1172/jci.insight.99762 PMC6124396 29925686

[34] Cotter DG Ercal B Huang X Leid JM d’Avignon DA Graham MJ Ketogenesis prevents diet-induced fatty liver injury and hyperglycemia. J Clin Invest (2014) 124:5175–90. 10.1172/JCI76388 25347470 PMC4348980

[35] Mederacke I Hsu CC Troeger JS Huebener P Mu X Dapito DH Fate tracing reveals hepatic stellate cells as dominant contributors to liver fibrosis independent of its aetiology. Nat Commun (2013) 4:2823. 10.1038/ncomms3823 24264436 PMC4059406

[36] Pradere JP Kluwe J De Minicis S Jiao JJ Gwak GY Dapito DH Hepatic macrophages but not dendritic cells contribute to liver fibrosis by promoting the survival of activated hepatic stellate cells in mice. Hepatology (2013) 58:1461–73. 10.1002/hep.26429 23553591 PMC3848418

[37] Puchalska P Martin SE Huang X Lengfeld JE Daniel B Graham MJ Hepatocyte-macrophage acetoacetate shuttle protects against tissue fibrosis. Cel Metab (2019) 29:383–98.e7. 10.1016/j.cmet.2018.10.015 PMC6559243 30449686

[38] van Hasselt PM Ferdinandusse S Monroe GR Ruiter JP Turkenburg M Geerlings MJ Monocarboxylate transporter 1 deficiency and ketone utilization. N Engl J Med (2014) 371:1900–7. 10.1056/NEJMoa1407778 25390740

[39] Luo X Li Z Chen L Zhang X Zhu X Wang Z Monocarboxylate transporter 1 in the liver modulates high-fat diet-induced obesity and hepatic steatosis in mice. Metabolism (2023) 143:155537. 10.1016/j.metabol.2023.155537 36933792

[40] Williamson DH. Mechanisms for the regulation of ketogenesis. Proc Nutr Soc (1981) 40:93–8. 10.1079/pns19810014 7010360

[41] Cotter DG Ercal B d'Avignon DA Dietzen DJ Crawford PA. Impairments of hepatic gluconeogenesis and ketogenesis in PPARalpha-deficient neonatal mice. Am J Physiol Endocrinol Metab (2014) 307:E176–185. 10.1152/ajpendo.00087.2014 24865983 PMC4101633

[42] Montagner A Polizzi A Fouché E Ducheix S Lippi Y Lasserre F Liver PPARα is crucial for whole-body fatty acid homeostasis and is protective against NAFLD. Gut (2016) 65:1202–14. 10.1136/gutjnl-2015-310798 26838599 PMC4941147

[43] Qi C Zhu Y Reddy JK. Peroxisome proliferator-activated receptors, coactivators, and downstream targets. Cel Biochem Biophys (2000) 32:187–204. 10.1385/cbb:32:1-3:187 11330046

[44] Sengupta S Peterson TR Laplante M Oh S Sabatini DM. mTORC1 controls fasting-induced ketogenesis and its modulation by ageing. Nature (2010) 468:1100–4. 10.1038/nature09584 21179166

[45] Feng J Qiu S Zhou S Tan Y Bai Y Cao H mTOR: a potential New target in nonalcoholic fatty liver disease. Int J Mol Sci (2022) 23:9196. 10.3390/ijms23169196 36012464 PMC9409235

[46] Gao Y Sheng X Tan D Kim S Choi S Paudel S Identification of histone lysine acetoacetylation as a dynamic post-translational modification regulated by HBO1. Adv Sci (2023) 10:e2300032. 10.1002/advs.202300032 PMC10477889 37382194

[47] Xie Z Zhang D Chung D Tang Z Huang H Dai L Metabolic regulation of gene expression by histone lysine β-hydroxybutyrylation. Mol Cel (2016) 62:194–206. 10.1016/j.molcel.2016.03.036 PMC5540445 27105115

[48] Shimazu T Hirschey MD Newman J He W Shirakawa K Le Moan N Suppression of oxidative stress by β-hydroxybutyrate, an endogenous histone deacetylase inhibitor. Science (2013) 339:211–4. 10.1126/science.1227166 23223453 PMC3735349

[49] Puchalska P Crawford PA. Metabolic and signaling roles of ketone bodies in health and disease. Annu Rev Nutr (2021) 41:49–77. 10.1146/annurev-nutr-111120-111518 34633859 PMC8922216

[50] Bendridi N Selmi A Balcerczyk A Pirola L. Ketone bodies as metabolites and signalling molecules at the crossroad between inflammation and epigenetic control of cardiometabolic disorders. Int J Mol Sci (2022) 23:14564. 10.3390/ijms232314564 36498891 PMC9740056

[51] Zhou T Cheng X He Y Xie Y Xu F Xu Y Function and mechanism of histone β-hydroxybutyrylation in health and disease. Front Immunol (2022) 13:981285. 10.3389/fimmu.2022.981285 36172354 PMC9511043

[52] Hwang CY Choe W Yoon KS Ha J Kim SS Yeo EJ Molecular mechanisms for ketone body metabolism, signaling functions, and therapeutic potential in cancer. Nutrients (2022) 14:4932. 10.3390/nu14224932 36432618 PMC9694619

[53] Asif S Morrow NM Mulvihill EE Kim KH. Understanding dietary intervention-mediated epigenetic modifications in metabolic diseases. Front Genet (2020) 11:590369. 10.3389/fgene.2020.590369 33193730 PMC7593700

[54] Xu BT Teng F Wu Q Wan S Li X Tan X Bdh1 overexpression ameliorates hepatic injury by activation of Nrf2 in a MAFLD mouse model. Cell Death Discov (2022) 8:49. 10.1038/s41420-022-00840-w 35115498 PMC8814004

[55] Chen Y Ouyang X Hoque R Garcia-Martinez I Yousaf MN Tonack S β-Hydroxybutyrate protects from alcohol-induced liver injury via a Hcar2-cAMP dependent pathway. J Hepatol (2018) 69:687–96. 10.1016/j.jhep.2018.04.004 29705237 PMC6098974

[56] Miyauchi T Uchida Y Kadono K Hirao H Kawasoe J Watanabe T Up-regulation of FOXO1 and reduced inflammation by β-hydroxybutyric acid are essential diet restriction benefits against liver injury. Proc Natl Acad Sci U S A (2019) 116:13533–42. 10.1073/pnas.1820282116 31196960 PMC6613133

[57] Hazem SH Hamed MF Saad MA Gameil NM. Comparison of lactate and β-hydroxybutyrate in the treatment of concanavalin-A induced hepatitis. Int Immunopharmacology (2018) 61:376–84. 10.1016/j.intimp.2018.06.026 29945025

[58] Moore MP Cunningham RP Davis RAH Deemer SE Roberts BM Plaisance EP A dietary ketone ester mitigates histological outcomes of NAFLD and markers of fibrosis in high-fat diet fed mice. Am J Physiology-Gastrointestinal Liver Physiol (2021) 320:G564–G572. 10.1152/ajpgi.00259.2020 PMC8238172 33501889

[59] Rushing KA Bolyard ML Kelty T Wieschhaus N Pavela G Rector RS Dietary ketone ester attenuates the accretion of adiposity and liver steatosis in mice fed a high-fat, high-sugar diet. Front Physiol (2023) 14:1165224. 10.3389/fphys.2023.1165224 37113697 PMC10128912

[60] Kim KE Jung Y Min S Nam M Heo RW Jeon BT Caloric restriction of db/db mice reverts hepatic steatosis and body weight with divergent hepatic metabolism. Sci Rep (2016) 6:30111. 10.1038/srep30111 27439777 PMC4954985

[61] Ravussin E Redman LM Rochon J Das SK Fontana L Kraus WE A 2-year randomized controlled trial of human caloric restriction: feasibility and effects on predictors of health span and longevity. The Journals Gerontol Ser A: Biol Sci Med Sci (2015) 70:1097–104. 10.1093/gerona/glv057 PMC4841173 26187233

[62] Lin AL Zhang W Gao X Watts L. Caloric restriction increases ketone bodies metabolism and preserves blood flow in aging brain. Neurobiol Aging (2015) 36:2296–303. 10.1016/j.neurobiolaging.2015.03.012 25896951 PMC4457572

[63] Ferguson BS Sahoo P McGrail E Francois A Stratton MS. Modestly increased incidence of ketosis in caloric restriction does not significantly alter the effects of caloric restriction. The J Nutr Health Aging (2022) 26:657–62. 10.1007/s12603-022-1815-7 35842755 PMC9704061

[64] Ravaut G Carneiro A Mounier C. Exploring the impacts of ketogenic diet on reversible hepatic steatosis: initial analysis in male mice. Front Nutr (2024) 11:1290540. 10.3389/fnut.2024.1290540 38577162 PMC10991688

[65] Luukkonen PK Dufour S Lyu K Zhang XM Hakkarainen A Lehtimäki TE Effect of a ketogenic diet on hepatic steatosis and hepatic mitochondrial metabolism in nonalcoholic fatty liver disease. Proc Natl Acad Sci U S A (2020) 117:7347–54. 10.1073/pnas.1922344117 32179679 PMC7132133

[66] Rector RS Uptergrove GM Morris EM Borengasser SJ Laughlin MH Booth FW Daily exercise vs. caloric restriction for prevention of nonalcoholic fatty liver disease in the OLETF rat model. Am J Physiology-Gastrointestinal Liver Physiol (2011) 300:G874–883. 10.1152/ajpgi.00510.2010 PMC3094141 21350190

[67] Dong F Zhang Y Huang Y Wang Y Zhang G Hu X Long-term lifestyle interventions in middle-aged and elderly men with nonalcoholic fatty liver disease: a randomized controlled trial. Sci Rep (2016) 6:36783. 10.1038/srep36783 27830836 PMC5103187

[68] Mardinoglu A Wu H Bjornson E Zhang C Hakkarainen A Räsänen SM An integrated understanding of the rapid metabolic benefits of a carbohydrate-restricted diet on hepatic steatosis in humans. Cel Metab (2018) 27:559–71.e5. 10.1016/j.cmet.2018.01.005 PMC6706084 29456073

[69] Tendler D Lin S Yancy WS Jr Mavropoulos J Sylvestre P Rockey DC The effect of a low-carbohydrate, ketogenic diet on nonalcoholic fatty liver disease: a pilot study. Dig Dis Sci (2007) 52:589–93. 10.1007/s10620-006-9433-5 17219068

[70] Newman JC Covarrubias AJ Zhao M Yu X Gut P Ng CP Ketogenic diet reduces midlife mortality and improves memory in aging mice. Cel Metab (2017) 26:547–57.e8. 10.1016/j.cmet.2017.08.004 PMC5605815 28877458

[71] Cunha GM Guzman G Correa De Mello LL Trein B Spina L Bussade I Efficacy of a 2-month very low-calorie ketogenic diet (VLCKD) compared to a standard low-calorie diet in reducing visceral and liver fat accumulation in patients with obesity. Front Endocrinol (Lausanne) (2020) 11:607. 10.3389/fendo.2020.00607 33042004 PMC7521128

[72] Nasser S Solé T Vega N Thomas T Balcerczyk A Strigini M Ketogenic diet administration to mice after a high-fat-diet regimen promotes weight loss, glycemic normalization and induces adaptations of ketogenic pathways in liver and kidney. Mol Metab (2022) 65:101578. 10.1016/j.molmet.2022.101578 35995402 PMC9460189

[73] Guo W Cao H Shen Y Li W Wang W Cheng L Role of liver FGF21-KLB signaling in ketogenic diet-induced amelioration of hepatic steatosis. Nutr Diabetes (2024) 14:18. 10.1038/s41387-024-00277-3 38609395 PMC11014968

[74] Badman MK Pissios P Kennedy AR Koukos G Flier JS Maratos-Flier E. Hepatic fibroblast growth factor 21 is regulated by PPARα and is a key mediator of hepatic lipid metabolism in ketotic states. Cel Metab (2007) 5:426–37. 10.1016/j.cmet.2007.05.002 17550778

[75] Newman JC Verdin E. Ketone bodies as signaling metabolites. Trends Endocrinol and Metab (2014) 25:42–52. 10.1016/j.tem.2013.09.002 24140022 PMC4176946

[76] Long F Bhatti MR Kellenberger A Sun W Modica S Höring M A low-carbohydrate diet induces hepatic insulin resistance and metabolic associated fatty liver disease in mice. Mol Metab (2023) 69:101675. 10.1016/j.molmet.2023.101675 36682412 PMC9900440

[77] Wei SJ Schell JR Chocron ES Varmazyad M Xu G Chen WH Ketogenic diet induces p53-dependent cellular senescence in multiple organs. Sci Adv (2024) 10:eado1463. 10.1126/sciadv.ado1463 38758782 PMC11100565

[78] Di Francesco A Di Germanio C Bernier M de Cabo R. A time to fast. Science (2018) 362:770–5. 10.1126/science.aau2095 30442801 PMC8504313

[79] Longo VD Mattson MP. Fasting: molecular mechanisms and clinical applications. Cel Metab (2014) 19:181–92. 10.1016/j.cmet.2013.12.008 PMC3946160 24440038

[80] Geisler CE Ghimire S Bogan RL Renquist BJ. Role of ketone signaling in the hepatic response to fasting. Am J Physiology-Gastrointestinal Liver Physiol (2019) 316:G623–G631. 10.1152/ajpgi.00415.2017 PMC6580236 30767679

[81] Gallage S Ali A Barragan Avila JE Seymen N Ramadori P Joerke V A 5:2 intermittent fasting regimen ameliorates NASH and fibrosis and blunts HCC development via hepatic PPARalpha and PCK1. Cell Metab (2024) 36:1371–93 e1377. 10.1016/j.cmet.2024.04.015 38718791

[82] Elsayed HRH El-Nablaway M Khattab BA Sherif RN Elkashef WF Abdalla AM Independent of calorie intake, short-term alternate-day fasting alleviates NASH, with modulation of markers of lipogenesis, autophagy, apoptosis, and inflammation in rats. J Histochem Cytochem (2021) 69:575–96. 10.1369/00221554211041607 34448436 PMC8427931

[83] Marinho Td. S Ornellas F Barbosa-da-Silva S Mandarim-de-Lacerda CA Aguila MB. Beneficial effects of intermittent fasting on steatosis and inflammation of the liver in mice fed a high-fat or a high-fructose diet. Nutrition (2019) 65:103–12. 10.1016/j.nut.2019.02.020 31079017

[84] Kim KE Shin HJ Ju Y Jung Y An HS Lee SJ Intermittent fasting attenuates metabolic-dysfunction-associated steatohepatitis by enhancing the hepatic autophagy-lysosome pathway. Nutrients (2023) 15:4574. 10.3390/nu15214574 37960230 PMC10649202

[85] Damasceno de Lima R Fudoli Lins Vieira R Rosetto Muñoz V Chaix A Azevedo Macedo AP Calheiros Antunes G Time-restricted feeding combined with resistance exercise prevents obesity and improves lipid metabolism in the liver of mice fed a high-fat diet. Am J Physiology-Endocrinology Metab (2023) 325:E513–E528. 10.1152/ajpendo.00129.2023 PMC10864020 37755454

[86] Wei X Lin B Huang Y Yang S Huang C Shi L Effects of time-restricted eating on nonalcoholic fatty liver disease: the TREATY-FLD randomized clinical trial. JAMA Netw Open (2023) 6:e233513. 10.1001/jamanetworkopen.2023.3513 36930148 PMC10024204

[87] Fu ZD Cai XL Yang WJ Zhao MM Li R Li YF. Novel glucose-lowering drugs for non-alcoholic fatty liver disease. World J Diabetes (2021) 12:84–97. 10.4239/wjd.v12.i1.84 33520110 PMC7807257

[88] Fougerat A Montagner A Loiseau N Guillou H Wahli W. Peroxisome proliferator-activated receptors and their novel ligands as candidates for the treatment of non-alcoholic fatty liver disease. Cells (2020) 9:1638. 10.3390/cells9071638 32650421 PMC7408116

[89] Zou W Zhang C Gu X Li X Zhu H. Metformin in combination with malvidin prevents progression of non-alcoholic fatty liver disease via improving lipid and glucose metabolisms, and inhibiting inflammation in type 2 diabetes rats. Drug Des Develop Ther (2021) 15:2565–76. 10.2147/DDDT.S307257 PMC8218939 34168429

[90] Pinyopornpanish K Leerapun A Pinyopornpanish K Chattipakorn N. Effects of metformin on hepatic steatosis in adults with nonalcoholic fatty liver disease and diabetes: insights from the cellular to patient levels. Gut and Liver (2021) 15:827–40. 10.5009/gnl20367 33820884 PMC8593497

[91] Tessari P Tiengo A. Metformin treatment of rats with diet-induced overweight and hypertriglyceridemia decreases plasma triglyceride concentrations, while decreasing triglyceride and increasing ketone body output by the isolated perfused liver. Acta Diabetol (2008) 45:143–5. 10.1007/s00592-008-0032-0 18357405

[92] Cuyas E Fernández-Arroyo S Buxó M Pernas S Dorca J Álvarez I Metformin induces a fasting- and antifolate-mimicking modification of systemic host metabolism in breast cancer patients. Aging (Albany NY) (2019) 11:2874–88. 10.18632/aging.101960 31076561 PMC6535060

[93] Qiu YY Zhang J Zeng FY Zhu YZ. Roles of the peroxisome proliferator-activated receptors (PPARs) in the pathogenesis of nonalcoholic fatty liver disease (NAFLD). Pharmacol Res (2023) 192:106786. 10.1016/j.phrs.2023.106786 37146924

[94] Souza-Mello V. Peroxisome proliferator-activated receptors as targets to treat non-alcoholic fatty liver disease. World J Hepatol (2015) 7:1012–9. 10.4254/wjh.v7.i8.1012 26052390 PMC4450178

[95] Shinozaki S Tahara T Miura K Kawarai Lefor A Yamamoto H. Pemafibrate therapy for non-alcoholic fatty liver disease is more effective in lean patients than obese patients. Clin Exp Hepatol (2022) 8:278–83. 10.5114/ceh.2022.120099 36683866 PMC9850303

[96] Honda Y Kessoku T Ogawa Y Tomeno W Imajo K Fujita K Pemafibrate, a novel selective peroxisome proliferator-activated receptor alpha modulator, improves the pathogenesis in a rodent model of nonalcoholic steatohepatitis. Sci Rep (2017) 7:42477. 10.1038/srep42477 28195199 PMC5307366

[97] Bian H Liu YM Chen ZN. New avenues for NASH therapy by targeting ACC. Cel Metab (2022) 34:191–3. 10.1016/j.cmet.2022.01.001 35108509

[98] Alkhouri N Lawitz E Noureddin M DeFronzo R Shulman GI. GS-0976 (Firsocostat): an investigational liver-directed acetyl-CoA carboxylase (ACC) inhibitor for the treatment of non-alcoholic steatohepatitis (NASH). Expert Opin Investig Drugs (2020) 29:135–41. 10.1080/13543784.2020.1668374 PMC7063378 31519114

[99] Calle RA Amin NB Carvajal-Gonzalez S Ross TT Bergman A Aggarwal S ACC inhibitor alone or co-administered with a DGAT2 inhibitor in patients with non-alcoholic fatty liver disease: two parallel, placebo-controlled, randomized phase 2a trials. Nat Med (2021) 27:1836–48. 10.1038/s41591-021-01489-1 34635855

[100] Zhang XJ Ji YX Cheng X Cheng Y Yang H Wang J A small molecule targeting ALOX12-ACC1 ameliorates nonalcoholic steatohepatitis in mice and macaques. Sci Transl Med (2021) 13:eabg8116. 10.1126/scitranslmed.abg8116 34910548

[101] Kim CW Addy C Kusunoki J Anderson NN Deja S Fu X Acetyl CoA carboxylase inhibition reduces hepatic steatosis but elevates plasma triglycerides in mice and humans: a bedside to bench investigation. Cel Metab (2017) 26:394–406.e6. 10.1016/j.cmet.2017.07.009 PMC5603267 28768177

[102] Gulette GA Hass DT Pandey K Zhang Q Han JY Engel A Reassessing retinal pigment epithelial ketogenesis: enzymatic assays for ketone body levels provide inaccurate results. Exp Eye Res (2024) 245:109966. 10.1016/j.exer.2024.109966 38857822 PMC12256115

[103] Hsia DS Grove O Cefalu WT. An update on sodium-glucose co-transporter-2 inhibitors for the treatment of diabetes mellitus. Curr Opin Endocrinol Diabetes and Obes (2017) 24:73–9. 10.1097/MED.0000000000000311 27898586 PMC6028052

[104] Yaribeygi H Maleki M Butler AE Jamialahmadi T Sahebkar A. New insights into cellular links between sodium-glucose cotransporter-2 inhibitors and ketogenesis. J Cell Biochem (2022) 123:1879–90. 10.1002/jcb.30327 36153819

[105] Hoong CWS Chua MWJ. SGLT2 inhibitors as calorie restriction mimetics: insights on longevity pathways and age-related diseases. Endocrinology (2021) 162. 10.1210/endocr/bqab079 33857309

[106] Wei Q Xu X Guo L Li J Li L. Effect of SGLT2 inhibitors on type 2 diabetes mellitus with non-alcoholic fatty liver disease: a meta-analysis of randomized controlled trials. Front Endocrinol (Lausanne) (2021) 12:635556. 10.3389/fendo.2021.635556 34220701 PMC8247927

[107] Capozzi ME Coch RW Koech J Astapova II Wait JB Encisco SE The limited role of glucagon for ketogenesis during fasting or in response to SGLT2 inhibition. Diabetes (2020) 69:882–92. 10.2337/db19-1216 32005706 PMC7171961

[108] Berger JH Matsuura TR Bowman CE Taing R Patel J Lai L Sodium-glucose co-transporter 2 inhibitors act independently of SGLT2 to confer benefit for heart failure with reduced ejection fraction in mice. bioRxiv (2024) 2004.2029:591665. 10.1101/2024.04.29.591665

[109] Al Batran R Gopal K Capozzi ME Chahade JJ Saleme B Tabatabaei-Dakhili SA Pimozide alleviates hyperglycemia in diet-induced obesity by inhibiting skeletal muscle ketone oxidation. Cel Metab (2020) 31:909–19.e8. 10.1016/j.cmet.2020.03.017 32275862

[110] Uehara K Sostre-Colón J Gavin M Santoleri D Leonard KA Jacobs RL Activation of liver mTORC1 protects against NASH via dual regulation of VLDL-TAG secretion and *de novo* lipogenesis. Cell Mol Gastroenterol Hepatol (2022) 13:1625–47. 10.1016/j.jcmgh.2022.02.015 35240344 PMC9046248

[111] Musso G Saba F Cassader M Gambino R. Diabetic ketoacidosis with SGLT2 inhibitors. BMJ (2020) 371:m4147. 10.1136/bmj.m4147 33184044

[112] Slade S Ashurst J. Diet-induced ketoacidosis in a non-diabetic: a case report. Clin Pract Cases Emerg Med (2020) 4:259–62. 10.5811/cpcem.2020.2.44736 32426688 PMC7220017

[113] Smolensky I Zajac-Bakri K Odermatt TS Brégère C Cryan JF Guzman R Sex-specific differences in metabolic hormone and adipose tissue dynamics induced by moderate low-carbohydrate and ketogenic diet. Sci Rep (2023) 13:16465. 10.1038/s41598-023-43587-9 37777528 PMC10542803

[114] Chaix A Deota S Bhardwaj R Lin T Panda S. Sex- and age-dependent outcomes of 9-hour time-restricted feeding of a Western high-fat high-sucrose diet in C57BL/6J mice. Cel Rep (2021) 36:109543. 10.1016/j.celrep.2021.109543 PMC8500107 34407415

[115] Eap B Nomura M Panda O Garcia TY King CD Rose JP Ketone body metabolism declines with age in mice in a sex-dependent manner. bioRxiv (2022) 2010.2005:511032. 10.1101/2022.10.05.511032

